# The Relationship between Mindfulness and Emotional Intelligence as a Protective Factor for Healthcare Professionals: Systematic Review

**DOI:** 10.3390/ijerph18105491

**Published:** 2021-05-20

**Authors:** Nerea Jiménez-Picón, Macarena Romero-Martín, José Antonio Ponce-Blandón, Lucia Ramirez-Baena, Juan Carlos Palomo-Lara, Juan Gómez-Salgado

**Affiliations:** 1 Centro Universitario de Enfermería Cruz Roja, University of Seville, 41009 Sevilla, Spain; nejipi@cruzroja.es (N.J.-P.); japonce@cruzroja.es (J.A.P.-B.); luraba@cruzroja.es (L.R.-B.); jucapa@cruzroja.es (J.C.P.-L.); 2 Department of Nursing, University of Huelva, 21007 Huelva, Spain; 3 Department of Sociology, Social Work and Public Health, Faculty of Labour Sciences, University of Huelva, 21007 Huelva, Spain; salgado@uhu.es; 4 Safety and Health Postgraduate Programme, Universidad Espíritu Santo, Guayaquil, Ecuador

**Keywords:** emotional intelligence, mindfulness, healthcare professionals, systematic review

## Abstract

Emotional intelligence is an essential trait and skill for healthcare professionals. Mindfulness meditation has proved to be effective in increasing the wellbeing of those who practice it, leading to better mental health, self-care and job satisfaction. This paper aims to identify the recent evidence on the relationship between mindfulness and emotional intelligence among healthcare professionals and students. A systematic review was conducted including the databases PubMed, Cinhal, PsycINFO and Web of Science. The main variables were emotional intelligence skills and mindfulness practice. Data were extracted according to the following outcomes: authors, year of publication, country, study design, participants, mindfulness training intervention, tools used in data collection and main results. The following inclusion criteria were applied: peer-reviewed articles; published in English or Spanish; published between 2010 and 2020; quantitative methodology; a study population of healthcare professionals or students; the relationship with the aim of the study. The Joanna Briggs Institute criteria were followed for assessing the methodological quality of the selected studies. Three researchers were involved in the review. After the selection process, 10 studies were selected out of the 197 references initially identified. These studies revealed a positive relationship between mindfulness and emotional intelligence, particularly the capacity to regulate emotions. Furthermore, mindfulness is negatively related to emotional exhaustion. Training interventions based on mindfulness have proved to be useful in promoting emotional balance, emotional awareness, emotional acceptance, emotion recognition, expressive suppression and a reduction in emotional exhaustion. This study could serve as a basis for further research on the benefits of emotional intelligence and practicing mindfulness for the bio-psycho-social welfare of healthcare professionals.

## 1. Introduction

Emotional intelligence (EI) is a type of social intelligence that includes the capacity for controlling one’s emotions as well as those of others, identifying them and using this information to guide thoughts and actions, promoting a creative thought process, redirecting attention towards priority problems, increasing motivation and allowing for flexible planning [1]. It is composed of four facets: the perception and expression of one’s own emotions and those of others, emotional assimilation, understanding emotions and the capacity to regulate emotions [2]. The World Health Organization considers EI to be one of the ten life skills that support people to act in an adaptable and positive manner [3].

In the context of healthcare, EI is taking on an increasingly relevant role. EI has been shown to positively influence healthcare professionals’ bio-psycho-social welfare, increasing their individual resilience, their perception of social support, empathy, job performance and satisfaction, and reducing stress [4]. The ability to manage emotions is a fundamental skill that should be developed by healthcare professionals as their work environment often entails a significant emotional burden [5]. Specifically, there is evidence linking EI with effective clinical decision-making [6], the occurrence or reduction of stress and burnout in a work environment [7,8,9], work engagement [10] and, by extension, with the institution’s general productivity, turnover rate, work absenteeism and patient satisfaction [8,11,12]. Moreover, there is ample evidence showing the benefits of health professionals’ EI while undertaking daily tasks and on the physical and emotional care patients receive [4,13,14]. EI has been identified as a predictor of professional success [15]. Similarly, patients also perceive that professionals with EI provide higher quality care, recognizing empathy, active listening and compassion as aptitudes related to EI [16].

The nature of healthcare professionals’ work entails a marked emotional intensity that can lead to emotional exhaustion. This is a feeling of prolonged physical and emotional exhaustion, due to stress, overload and the challenging emotional job demands of care itself [17]. Healthcare professionals with emotional exhaustion have lower energy levels, struggle to face the tasks their job demands and have fewer opportunities for positive experiences in their work [18]. The emotional exhaustion has negative consequences on both the professionals’ mental health [19] and the quality of the care their patients receive, as the exhaustion is reflected in worse job performance and efficiency of care they provide [20]. Furthermore, emotional exhaustion has been linked to increased turnover intentions [21] and conflicts both among healthcare professionals and towards patients [22]. Emotional exhaustion is in part due to the intense emotional demands of caring, therefore professionals could become more resilient to such demands by developing skills such as EI [18].

EI has been described as a trait and a skill; for the purpose of this review, the authors consider EI as a skill that can be trained [8]. Many studies emphasize the importance of offering education in EI both to students and professionals, highlighting that the best moments for EI training are in childhood, higher education and adult life, as a part of continuous professional development [18]. Training in EI increases the bio-psycho-social welfare of healthcare professionals, as well as benefitting their clinical practice, the satisfaction of patients, and the institution for which they work [11,12,13].

Mindfulness meditation is an effective way of training EI. Mindfulness is a form of meditation based on the ability to bring one’s attention to what they are currently experiencing in the present moment, accepting it without judgement and identifying the sensations, emotions and thoughts [23]. This meditation is used as a therapeutic psychological treatment with positive results, reducing levels of anxiety, depression and stress, and alleviating physical symptoms such as pain as well as vulnerability due to psychiatric illnesses [24,25,26,27,28].

The relevance of the social context and emotional health for practicing healthy behaviors and improving fitness and wellbeing among healthcare workers has been studied [29]. Pronk et al. found that employees’ adherence to an optimal lifestyle such as physical activity, non-smoking, teetotalism and daily consumption of fruits or vegetables, was associated with significantly higher positive emotional health [30]. Hunter explains how professionals can obtain benefits from practicing this meditation [31] to gain greater control of their thoughts. This translates to a mental state of tranquility and calm, which provides them with perspective; these in turn are key elements for improving the care of patients and interprofessional communication. There is evidence of the impact and effectiveness of mindfulness for healthcare professionals, resulting in the reduction of stress and the promotion of self-compassion and self-care [32,33,34,35], the reduction of emotional exhaustion and the incidence of burnout [36,37], the improvement of wellbeing and mental health, and decreased anxiety and depression [38], with the same results being obtained in students [39,40]. All of this suggests that mindfulness could be a link between professionals’ clinical practice and their emotional management, helping them to maintain their own psychological health [32,39], while also resulting in direct benefits for patients and the healthcare system [41].

Despite the above, the relationship between practice and training in EI skills and mindfulness has been little studied among health professionals. As professionals who show a combination of technical skills and high levels of EI can provide more humanized health care [12], it would be convenient to offer interventions, programs or training based on mindfulness to healthcare professionals, in order to promote and develop their EI [42,43,44]. This paper aims to identify the recent evidence on the relationship between mindfulness and emotional intelligence among healthcare professionals and students.

## 2. Materials and Methods

To meet the objective of the study, a systematic review was conducted following the Preferred Reporting Items for Systematic Reviews and Meta-Analyses (PRISMA) guidelines [45]. The research protocol was not previously registered. The research question for this review was Is there a relationship between mindfulness practice and emotional intelligence skills among healthcare professionals and students? To facilitate the electronic search, it was structured according to the PICO model [46]. P (population) referred to healthcare professionals, I (intervention) referred to mindfulness practice, C (comparison intervention) refereed to no mindfulness practice and O (clinical outcome) referred to EI skills.

The search was carried out including the following databases: PubMed, Cinhal, PsycINFO and Web of Science. The search strategy was built up combining MeSH terms (emotional intelligence, emotional regulation, mindfulness, meditation, nurses, and physicians) and search words (emotional expression and healthcare professionals); in order to obtain a wider scope of records. The final search strategy was exactly as follows: (“emotional intelligence” OR “emotional regulation” OR “emotional expression”) AND (mindfulness OR meditation) AND (health professionals OR nurses OR physicians). The same search strategy was used in all the databases.

The search was conducted in October 2020. The following inclusion criteria were applied: peer-reviewed articles; published in English or Spanish; published between 2010 and 2020; quantitative methodology; a study population of healthcare professionals or students; consideration of EI as a skill; the relationship with the aim of the study (mindfulness and EI skills). The exclusion criteria included duplicate studies and those without access to the full-text version. Qualitative studies were excluded to obtain results that analyze the relationship between mindfulness and emotional intelligence measured with validated instruments. The search was limited to studies published from 2010 to 2020 as recent scientific publications render others previously published obsolete [47]. In addition, the obsolescence of older literature becomes evident due to the constant developments within this field. Relevancy was also measured through the citations of the articles, and articles published in the past ten years were cited the most [48].

In order to control biases, the methodological quality of the included studies was evaluated using the tool provided by the Joanna Briggs Institute for analytical cross-sectional studies [49]. This tool consists of eight items that allow for the estimation of the extent to which the reviewed studies have controlled for possible biases in their research, and thus to assess their inclusion in the study. Two reviewers assessed the selected studies independently following the assessment tool items. Each item checked on an element of bias and could be scored as Yes, No, Unclear, or Not applicable. They then shared their evaluations and resolved any differences through consensus. The research team agreed that studies that only obtained a negative appraisal in two or fewer items were included in the review.

The information from the selected studies was extracted using ad-hoc tables designed by the authors, which included the following sections: authors, year of publication, country, study design, participants, mindfulness training intervention, variables, tools used in data collection and main results. This data organization allowed for a qualitative and descriptive description of the results. Data synthesis was also performed independently by the researchers. Both conducted a thematic analysis of the results of the reviewed articles and categorized according to the objectives of the study: the relationship between mindfulness and EI, or with each of the skills it entails.

Two researchers independently carried out the search process, selection, evaluation, extraction, analysis and categorization of the results. They were PhD healthcare professionals and had strong experience in conducting systematic reviews previously. They were also familiar with the Joanna Briggs Institute assessment tool. Their findings were compared and contrasted, and any discrepancies were resolved through consensus, referring to a third reviewer when necessary. Researchers agreed the outcome from the review, so the third researcher’s intervention was not required.

## 3. Results

**Figure 1:**
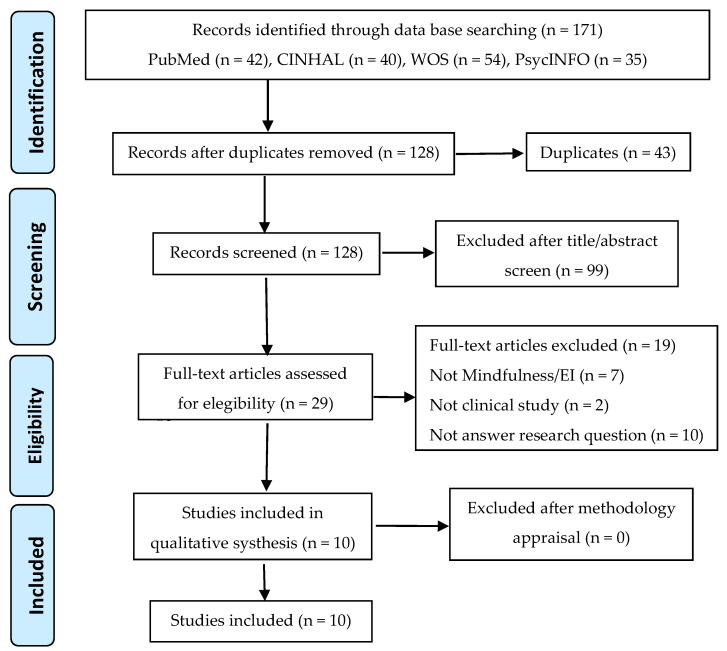
Flowchart of the selection process according to the Preferred Reporting Items for Systematic Reviews and Meta-Analyses (PRISMA).

The initial electronic search yielded 197 references. After applying the inclusion and exclusion criteria, and following the selection and screening process, 10 studies were finally selected (Figure 1).

The included studies were mainly published in the last five years (90%, n = 9). As to the design, they were predominantly cross-sectional studies (60%, n = 6), along with pretest-posttest studies (40%, n = 4). With regard to the study samples, most focused on nurses (60%, n = 6) compared to other healthcare professionals, and on professionals (60%, n = 6) as opposed to university students. As for the tools used for measuring mindfulness skills, the reviewed studies used the Mindfulness Attention Awareness Scale (30%, n = 3), the Five-Facet Mindfulness Questionnaire (20%, n = 2) and the Kentucky Inventory of Mindfulness Skills (10%, n = 1). The instruments used to assess emotional intelligence competencies were more varied, with the most frequently used being the Trait Emotional Intelligence Questionnaire (40%, n = 4). No consensus was found regarding the duration of mindfulness needed to protect against stress and burnout among the reviewed studies, ranging from 9 h to 14 min per day. Regarding methodological quality, all of the studies that were evaluated were included in the review. As is shown in Table 1, the least addressed aspect was the management of confounding factors.

Table 2 gathers the main findings of the reviewed studies related to this review’s objective [50,51,52,53,54,55,56,57,58,59].

The reviewed studies showed a relationship between mindfulness and the ability to regulate one’s emotions [52,58], emotional exhaustion [57] and EI [53,59]. The training interventions based on mindfulness have been shown to promote positive emotional balance among the participants [55], as well as emotional awareness [54], emotional acceptance, emotion recognition, identifying one’s own emotions, expressive suppression [56] and the reduction of emotional exhaustion [54]. With regard to EI, the reviewed studies have described interventions based on mindfulness training that have increased participants’ EI [50,51]. Other studies have identified an increase in EI after the intervention, although they did not achieve statistical significance [54,55].

## 4. Discussion

The results of the reviewed studies showed a relationship between mindfulness and EI skills, as well as an increase in EI after training interventions based on mindfulness. These results are consistent with other training interventions that have obtained positive results in their participants’ EI development [60]. The satisfactory experiences of EI training for healthcare professionals have taken place in a wide range of contexts, ranging from intensive care [61] to long-term care in nursing homes [62], including clinical areas [63,64,65]. Indeed, EI training has not only increased these skills in healthcare professionals but has also been associated with the patients’ quality of life [63], professionals’ job performance and job retention [64], their perceived state of health [61] and patient satisfaction [64].

Likewise, mindfulness training has been shown to provide healthcare professionals with psychological wellbeing by reducing stress, anxiety and depression [40,66,67,68], and by improving their mood [66]. There is evidence that healthcare professionals who have undergone mindfulness training have improved their quality of life [35,67], wellbeing [40], self-compassion [35] and perception of self-efficacy [40,66]. Previous studies indicated that learning mindfulness provides healthcare professionals with useful work skills such as empathy [40,66], concentration [35] and awareness of the present moment [35,67]. Furthermore, in accordance with the results of the reviewed studies, mindfulness training reduces burnout [40,67], which can manifest as emotional exhaustion, depersonalization and a reduced sense of personal accomplishment [68]. The reduction of burnout leads to a better performance at work, which is reflected in better communication with colleagues and patients, higher sensitivity to patients’ experiences, clearer analysis of complex situations and emotional regulation in stressful contexts [38].

According to the studies included in this review, mindfulness has been identified as an efficient practice for improving the perception and expression of one’s emotions, emotional assimilation or facilitation, and emotional understanding and regulation. Healthcare professionals face highly intense emotional challenges on a daily basis, as they witness human suffering and deal with patients’ anxiety and negative feelings; in addition to their work overload and interpersonal conflicts within their team [69]. In the words of Botha et al., the inherent duties of health professionals, such as managing pain, loss, emotional discomfort, end-of-life care and supporting family members, contribute to their emotional burden [35]. Because of this, they need to find a balance between their emotional investment in patients and detachment, which allows them to effectively respond to both the demands of the organization as well as the patients’ needs [70]. According to Wu et al., mindfulness promotes a positive attitude amongst healthcare professionals toward the do-not-resuscitate signature, and results in better care for terminally ill patients as it enhances their confidence to discuss end-of-life care decisions with patients and their families [71].

Emotional labor can be defined as the effort to control one’s own emotions to give an effective response to the needs of others, while still caring for oneself [72]. The emotional demands of managing one’s own emotions as well as those of others can have a negative influence on healthcare professionals’ health, wellbeing and job performance, leading to high levels of stress and burnout [73,74]. Long-term care workers prefer coping strategies such as mindfulness, meditation and solitary recreation, as well as drawing on social support, as a sources of stress relief [75]. It has been shown that practicing mindfulness reduces professionals’ emotional overload and burnout, while also enhancing positive cognitive retraining [37]. Mindfulness strategies and nature therapy (immersing oneself in nature using all five senses) have been suggested as useful techniques to promote resilience, lessen burnout and to heal oneself, for healthcare providers in rural environments [76]. Furthermore, mindfulness has been positively linked to the capacity for withstanding uncomfortable emotions and feelings, the reduction in the impact of harmful emotional events and lower emotional reactivity [77]. In this regard, previous studies have identified an association between mindfulness and self-compassion [78]. Self-compassion has been described as a feeling of kindness and empathy towards one’s own suffering or failure, which encourages understanding rather than self-criticism and punishment [79]. Some authors consider self-compassion to be a necessary attitude for allowing healthcare professionals to provide humanized care. Having compassion for others entails having self-compassion [80]. The awareness of one’s own suffering makes it easier to recognize the suffering of others and to give a significant response to alleviate it [81]. Therefore, as the practice of mindfulness develops self-compassion in healthcare professionals, it promotes attitudes of empathy and concern for patients’ feelings, which leads to a higher quality of care [80].

Lastly, the articles included in this review highlighted an increase in personal resilience among those healthcare professionals who have undergone mindfulness training. This resilience has been described as the capacity to recover from or cope with adverse circumstances [82]. Previous experiences of mindfulness training interventions have proved to be effective in producing a significant improvement in healthcare professionals’ capacity for resilience [83]. The practice of mindfulness has previously been associated with healthcare professionals’ personal resilience and self-compassion [84]. It has been identified that an increase in resilience can help them lessen the negative impact of their demanding jobs, reducing emotional exhaustion, increasing their commitment to their work and improving their performance when facing challenges in their workplace [85]. Mindfulness has also been identified as a protective factor against the stress caused by emotional labor [69].

This review is subject to the limitations associated with its methodology, such as publication and selection biases. In order to minimize the latter, the selection process was carried out by independent researchers. The possible biases of the reviewed studies must also be taken into account. To control for these, the studies’ methodological quality was evaluated before they were included in the review. The establishment of a quantitative methodology as one of the inclusion criteria has meant the exclusion of qualitative studies that may have contributed additional information towards the review’s objectives. Despite these limitations, the authors trust the association between EI and mindfulness that has been confirmed in this review.

## 5. Conclusions

This study has reviewed the relationship between the practice of mindfulness meditation and EI, as well as each of the skills that it comprises. EI in general was shown to increase significantly after practicing mindfulness, as did the perception and expression of emotions, and emotional regulation. The results regarding emotional assimilation and understanding have shown that both increase after practicing this type of meditation.

This review entails several important implications, as the development of EI in healthcare professionals can lead to an increase in their capacity to manage emotions, and this in turn leads to an increase in the quality of care they provide, their resilience, empathy and job satisfaction. Moreover, the practice of mindfulness provides useful tools that can be used by professionals not only in developing their EI but also in managing stressful factors.

Mindfulness meditation, as a way to promote the development of EI, is an innovative technique whose efficacy and benefits in different work contexts have already been demonstrated. Due to the diversity of available interventions, further research is necessary in order to determine which is most effective.

This study can be used as a foundation for future research on the benefits of developing EI, and also investigating the strategies to develop EI, with one of them being mindfulness, as well as the benefits that practicing mindfulness can have on the bio-psycho-social sphere of professionals, regardless of their field of work.

## Figures and Tables

**Table 1:** Critical appraisal of reviewed studies.

Assessing Item	Vongareesawat et al. 2012	Snowden et al. 2015	Dubert et al. 2016	Jacobs et al. 2016	Orellana et al. 2017	Kelm et al. 2018	Lamothe et al. 2018	Salvarani et al. 2019	Salvarani et al. 2020	Xie et al. 2020
Were the criteria for inclusion in the sample clearly defined?	Y	Y	Y	Y	Y	Y	Y	Y	Y	Y
Were the study subjects and the setting described in detail?	Y	Y	Y	Y	Y	Y	Y	Y	Y	Y
Was the exposure measured in a valid and reliable way?	Y	Y	Y	Y	Y	Y	Y	Y	Y	Y
Were objective, standard criteria used for measurement of the condition?	Y	Y	Y	Y	Y	Y	Y	Y	Y	Y
Were confounding factors identified?	Y	Y	Y	Y	Y	Y	Y	Y	Y	N
Were strategies to deal with confounding factors stated?	Y	Y	N	Y	N	Y	N	N	Y	Y
Were the outcomes measured in a valid and reliable way?	Y	Y	Y	Y	Y	Y	Y	Y	Y	Y
Was appropriate statistical analysis used?	Y	Y	Y	Y	Y	Y	Y	Y	Y	Y
Overall appraisal	I	I	I	I	I	I	I	I	I	I

Possible scores: Y = yes, N = no, U = unclear, N/A = not applicable, I = include, E = exclude, S = seek for further info.

**Table 2:** Summary of reviewed studies.

Authors Year Country	Design	Participants	Intervention	Variables (Assessment Tool)	Findings
Vongareesawat et al. [50] 2012. Thailand.	Pretest post-test (control group)	N = 26 psychiatric nurses	Mindfulness training 9 h/day 5 days/week 8 weeks	E Intelligence (TEISTTP)	EI scores significantly higher immediately after intervention (F = −2.13 *p* = 0.022) and at the one-month follow-up (F = −1.78 *p* = 0.044).
Snowden et al. [51] 2015. UK.	Cross-sectional	N = 870 nursing and midwifery students	Mindfulness training	E Intelligence (TEIQue, SEIS)	No significant differences in TEIQ-SF scores after mindfulness training (U = 22,980, z = 0.864, *p* = 0.388). Median SEIS significantly different according to mindfulness training (U = 25,115.5, z = 2.05, *p* = 0.039).
Dubert et al. [52] 2016. USA.	Cross-sectional	N = 80 nursing students	N/A	Dispositional mindfulnes (MAAS) E regulation (ERQ)	Positive correlation between MAAS and ERQ-R (r = 0.1905; *p* = 0.045). Direct effect with MAAS on ERQ-R (γ11 = 0.292 ± 0.158, *p* = 0.034)
Jacobs et al. [53] 2016. Germany.	Cross-sectional	N = 427 occupational therapists	N/A	Skills of mindfulness (KIMS) E Intelligence (TEIQue)	Trait E Intelligence correlated with skills of mindfulness observed (r = 0.19, *p* < 0.001), describe (r = 0.54, *p* < 0.001), act with awareness (r = 0.45, *p* < 0.001) and accept without judgment (r = 0.55, *p* < 0.001). Mindfulness facets explained 50.2% of variance of emotional intelligence [F(5,421) = 84.73, *p* < 0.001]
Orellana et al. [54] 2017. Germany.	Pretest post-test	N = 28 palliative healthcare professionals	Meditation training 2 h session 10 weeks	E regulation (ERSQ-27) Burnout (MBI)	E exhaustion decreased after meditation (t = −3.13, *p* < 0.005, d = 0.41) and E awareness increased (t = −2.87, *p* = 0.008, d = 0.45) as well as resilience (t = −2.47, *p* = 0.021, d = 0.43). E regulation skills increased but not significantly (t = −1.85, *p* > 0.05, d = 0.24).
Kelm et al. [55] 2018. USA.	Pretest post-test	N = 24 ICU health professionals	Mindfulness meditation 7 min twice/day or 14 min/day 4-week	CPR performance E intelligence (TEIQue) E balance (PANAS)	More positive E balance after intervention (Mean difference = 0.33, Cohen’s d = 0.43, *p* = 0.02). E intelligence improvement was not significant (Mean difference = 4, Cohen’s d = 0.28, *p* = 0.16).
Lamothe et al. [56] 2018. Canada.	Pretest post-test	N = 37 12 healthcare students 25 healthcare professionals	Mindfulness program 2 h/week 8 weeks 1 full-day retreat.	Dispositional mindfulness (MAAS) E competence (PEC, ERQ, GERT) E acceptance (AAQ-II)	Students showed significant differences after the program in E acceptance [d = 1.39; t(11) = 4.81, *p* < 0.001], emotion recognition [d = 1.20; t(11) = 4.14, *p* < 0.01], identifying own emotions [d = 0.77; t(11) = 2.67, *p* < 0.05], expressive suppression, [d = 0.73; t(11) = 2.53, *p* < 0.05].
Salvarani et al. [57] 2019. Italy.	Cross-sectional	N = 97 emergency nurses	N/A	Dispositional mindfulness (FFMQ) Ability to regulate emotions (DERS) Burnout (MBI)	Significant negative correlation between E exhaustion and regulation of emotions and dimensions of FFQM (*p* < 0.05) FFMQ subscales explained the 23.6% of the E exhaustion variance
Salvarani et al. [58] 2020. Italy.	Cross-sectional	N = 622 nursing students	N/A	Dispositional mindfulness (FFMQ) Ability to regulate emotions (DERS)	Positive correlation between mindfulness and all dimensions of the ability to regulate emotions (*p* < 0.05).
Xie et al. [59] 2020. China.	Cross-sectional	N = 883 ICU nurses	N/A	Dispositional mindfulness (MAAS) E Intelligence (EI) Burnout (MBI)	Association between mindfulness, E intelligence, E exhaustion, depersonalization, and personal accomplishment (*p* < 0.001). E intelligence partially mediates the relationships between mindfulness and E exhaustion (total effect −0.492, *p* = 0.011) and depersonalization (total effect −0.633, *p* = 0.018).

E = Emotional; FFMQ = Five-Facet Mindfulness Questionnaire; DERS = Difficulties in Emotion Regulation Scale; MAAS = Mindful Attention Awareness Scale; ERQ = Emotion Regulation Questionnaire; MBI = Maslach Burnout Inventory; TEIQue = Trait Emotional Intelligence Questionnaire; SEIS = Emotional Intelligence Scale; TEISTTP = Thai Emotional Intelligence Screening Test for the Thai Population; PEC = Profile of Emotional Competence; AAQ-II = Acceptance and Action Questionnaire-II; ERQ = Emotion Regulation Scale; GERT = Geneva Emotion Recognition Test; EI = Emotional Intelligence Scale; KIMS = Kentucky Inventory of Mindfulness Skills; MHB = Multiple Health Behavior Questionnaire; PANAS = positive and negative affect Schedule; ERSQ-27 = Emotion Regulation Skills Questionnaire; CPR = cardiopulmonary resuscitation.
